# Should Complex Cognitive Functions Be Mapped With Direct Electrostimulation in Wide-Awake Surgery? A Commentary

**DOI:** 10.3389/fneur.2021.721038

**Published:** 2021-08-26

**Authors:** Emmanuel Mandonnet

**Affiliations:** ^1^Hôpital Lariboisière, Paris, France; ^2^INSERM U1127 Institut du Cerveau et de la Moelle épinière (ICM), Paris, France; ^3^Université de Paris, Paris, France

**Keywords:** meta-networks, glioma surgery, awake surgery, brain mapping, cognitive outcome

In a recent opinion (1), Herbet thoughtfully questioned the conceptual relevance of mapping complex cognitive functions with direct electrical stimulation during awake surgery.

His argumentation followed two lines of reasonings.

First, it might be that it is methodologically impossible to map of these functions intraoperatively, due to both electrophysiological and behavioral limitations. On an electrophysiological point of view, a single, focal, low-intensity input gate into the brain circuits (2) might be insufficient to cause an imbalance of the whole brain dynamics, up to the point that it would cause a behavioral impairment of complex cognitive functions. This view is grounded in the meta-networking theory of these complex cognitive functions: the goal-directed, flexible, context-sensitive behavior of humans would implicate a short-lived (metastable) spatio-temporal reconfiguration/cooperation between several domain-specific networks (3). Apart from the methodological inadequacy of using low-intensity focal electrical stimulation to disturb meta-networks, Herbet also argues that it could be impossible to design a behavioral task that would enable to monitor such functions, given the intraoperative constraints. In particular, electrical stimulation should be applied no longer than a few seconds, and such a short period of time considerably limits the possibilities. Finally, the well-known interindividual anatomical variability of modular and domain-specific functional areas could be even greater for the dynamics of meta-networking, and such a variability would make the interpretation and reproducibility of stimulation-induced disturbances very difficult.

Second, Herbet suggests that there is maybe no need to pay attention to these complex cognitive functions, because they would be much more resilient to damage than are modular or domain-specific functions. Indeed, as they emerge from a widespread meta-networking brain, these functional systems would exhibit a high degree of plasticity, and no long-term dysfunction would be observed after a brain tumor resection.

In this commentary, I would like to both complement and nuance this opinion.

The hypothesis put forward by Herbet is somehow supported by a recent study (4), demonstrating the rarity of self-reported effects when stimulating areas belonging to hetero-modal and trans-modal areas, strongly contrasting with unimodal areas: the gradient in the frequency of self-reported effects clearly follows the principal gradient of the cortical hierarchy found by Margulies et al. (5). It is true that a single entry point of stimulation might not be the optimal way to unveil areas at the distal end of the cortical hierarchy. However, it should be noted that, in a recent study, multisite stimulation unmasked language sites that were not detected by mono-site stimulation (6). Such scheme of stimulation could thus be used to desynchronize a set of areas, helping to identify a meta-network. More generally, mathematical tools of network dynamics, like network controllability (7), could guide the elaboration of even more sophisticated stimulation protocols, possibly better suited to induce meta-network dysfunction.

The issue of the task design is a serious one, as it is absolutely right that the limited timing (a few seconds) puts a very strong constraint. However, in the aforementioned study (4), self-reported effects were also noted, albeit rarely, after stimulation of hetero/trans-modal areas. When these rare effects occurred, they were quite complex (for example, “Patient describes a negative emotional feeling, seemingly localized in the chest”), suggesting that they might have been indeed generated from a meta-networking dysfunction. Hence, assessing detailed introspection could provide a mean to overcome the constraints imposed by the intraoperative setting when designing a task targeting the meta-networking activity. This is also in line with the recent proposal to monitor patient's self-evaluation of his performance after each task trial, in addition to the performance itself (8). This additional monitoring is relatively easy to implement, but, admittedly, its value is currently not well-established.

We fully concur with the author that the high interindividual variability of meta-networks anatomical implementation might greatly complicate the reproducible identification of “complex cognitive areas.” And in the same perspective, the well-known non-reproducibility on a trial-by-trial basis of mono-site stimulation (9) might also fit with the idea that the dynamics of meta-networking is highly variable, even at the scale of a single brain: from one trial to another, the brain dynamics might have followed a different trajectory, explaining why two consecutive stimulations of the same site do not always produce the same behavioral dysfunction, even for such a simple task as picture naming.

We thus claim that it is possible to envision solutions that would enable to interfere intra-operatively with the meta-networks of complex cognition. But, as argued by Herbet in the second part of his argumentation, the most important practical clinical question is rather the following: assuming we are able to identify intraoperatively a complex cognitive function site, should the surgeon stop the resection at this level or should he push further the resection, betting on late recovery thanks to the virtue of plasticity?

We fully agree with the idea that there exists a gradient of plasticity from unimodal/modular areas to modality-specific or domain-specific networks. Let me consider the example of executive functions, although the same would apply to social cognition (10–12). Recently, several groups independently reported that immediate postoperative deficits in executive functions are commonly observed in low-grade glioma patients in whom the resection was performed without intraoperative monitoring of these executive functions (13–20). Importantly, these series also demonstrated that only a relatively small fraction of patients remained definitely impaired. This means that if executive functions would have been systematically mapped and preserved, the resection would have been stopped prematurely in a large proportion of patients, with a loss of chance regarding oncological benefit. This contrasts with modular input-output areas, for which we are 100 % sure that their resection would result in permanent deficits, and thus should be preserved systematically. To address this issue, two broad categories of solutions have been implemented. Skrap et al. proposed an “*ad hoc*” pragmatic approach: “The limit used to stop surgery temporarily or definitively coincided with a decrease in performance to 70% of baseline levels. The 30% threshold was established by examining a preliminary group of patients in whom we tested the feasibility of our technique before actually starting the present study. Thus, this threshold was correlated to follow-up performance” (21). Other authors preferred to follow a “mechanistic” approach: they attempted first to retrospectively determine the precise networks of the brain the resection of which induced deficits, and restricted the use of intraoperative executive functions tasks to these locations (14, 18, 22, 23). In the future, there is a strong call for developing new methods (24), relying on machine learning algorithms, that would enable to predict, from a set of preoperative personalized predictors reflecting individual brain functioning, which function would be at risk in an individual patient and would thus deserve to be mapped intraoperatively.

In fact, the degree of plasticity of a modality-specific/domain-specific network cannot be defined in an absolute manner. The measurement of the plasticity of a network is always relative to the task measuring its function. For example, when Herbet argues that “despite the central role of the anterior temporal structures within the semantic memory network, unilateral damage of this region in various pathophysiological conditions (including glioma) does not result in the severe impairments of semantic representations it might be expected,” this statement implicitly depends on which task is used to assess semantic knowledge. While it is true that a basic non-verbal semantic association task like the pyramid-palm-tree test fails to detect any impairment after anterior temporal resections (25), long-term deficits in proper names retrieval have been reported (26). This is likely because it is only from the left side that the lexical store can access the bilateral representations of conceptual entities, shared by the left and right anterior temporal structures. Similarly, should we measure semantic knowledge by metrics estimated on full semantic graphs (27), new kind of deficits would be likely unveiled.

To which extent plasticity is even more efficient for complex cognition remains unknown. As acknowledged in the conclusion by the author, “no works have currently assessed in a longitudinal manner if complex and flexible cognitions/behaviors are impaired following neurosurgical procedures (knowing that they are not probed with routine neuropsychological tasks) and if these possible impairments are disabling in daily life.”

To go further, it would have been informative to illustrate a little bit more what is exactly meant by “complex and flexible cognition/behavior.” The unique example given by Herbet is learning abilities, raising the question whether patients do suffer (or not) postoperatively from learning problems. And indeed, to the best of my knowledge, the literature on this question is virtually non-existent. Thinking about other examples, should we include in this category the processes allowing to understand metaphors? This ability necessitates the coordination of several networks: semantic knowledge, language, attention, working memory, executive system. Importantly, plasticity does not fully protect metaphors comprehension from surgical damage: studies have reported postoperative deficits after resection in the right hemisphere (21). And interestingly, it seems feasible to test intraoperatively the comprehension of metaphors (21).

Last but not least, it should be kept in mind that, even if plasticity allows patients to recover complex cognition performances close to normal, the resulting suboptimal brain dynamics comes with a price to pay: patients are both slower and more fatigable. These two downsides should not be overlooked: it has been demonstrated that both slowness and fatigue correlate with employment outcomes (28, 29). Hence, in the context of surgery for incidentally discovered glioma (30)—and even so in the context of a screening policy (31)—, great care should be devoted to preserve as much as possible a fully normal functioning of meta-networks.

In summary, Herbet opened a very interesting debate regarding what could be the limitations of cognitive monitoring in an awake patient for preserving the integrity of its complex cognitive functions. Such a topic might seem a little bit premature, considering that we still have a lot to learn regarding modality-specific/domain-specific functions so that we could better predict in which patient and at which location which function should be mapped intraoperatively. Nevertheless, efforts should be devoted to design new tasks in order to better explore complex cognitive functions extra-operatively, to evaluate how they might be impacted by surgical resection, and to imagine new pre- and intra- operative methods to offer the patients the maximal chances to preserve an efficient functioning of their meta-networks.

## Author Contributions

The author confirms being the sole contributor of this work and has approved it for publication.

## Conflict of Interest

The author declares that the research was conducted in the absence of any commercial or financial relationships that could be construed as a potential conflict of interest.

## Publisher's Note

All claims expressed in this article are solely those of the authors and do not necessarily represent those of their affiliated organizations, or those of the publisher, the editors and the reviewers. Any product that may be evaluated in this article, or claim that may be made by its manufacturer, is not guaranteed or endorsed by the publisher.
